# Motor imagery during action observation modulates automatic imitation effects in rhythmical actions

**DOI:** 10.3389/fnhum.2014.00028

**Published:** 2014-02-18

**Authors:** Daniel L. Eaves, Lauren Haythornthwaite, Stefan Vogt

**Affiliations:** ^1^Sport and Exercise Science Section, School of Social Sciences and Law, Teesside UniversityMiddlesbrough, UK; ^2^Department of Psychology, Fylde College, Lancaster UniversityLancaster, UK

**Keywords:** motor simulation, mirror neurons, joint action, mental practice, video therapy, observational learning, stroke rehabilitation, movement demonstrations

## Abstract

We have previously shown that passively observing a task-irrelevant rhythmical action can bias the cycle time of a subsequently executed rhythmical action. Here we use the same paradigm to investigate the impact of different forms of motor imagery (MI) during action observation (AO) on this automatic imitation (AI) effect. Participants saw a picture of the instructed action followed by a rhythmical distractor movie, wherein cycle time was subtly manipulated across trials. They then executed the instructed rhythmical action. When participants imagined performing the instructed action in synchrony with the distractor action (AO + MI), a strong imitation bias was found that was significantly greater than in our previous study. The bias was pronounced equally for compatible and incompatible trials, wherein observed and imagined actions were different in type (e.g., face washing vs. painting) or plane of movement, or both. In contrast, no imitation bias was observed when MI conflicted with AO. In Experiment 2, motor execution synchronized with AO produced a stronger imitation bias compared to AO + MI, showing an advantage in synchronization for overt execution over MI. Furthermore, the bias was stronger when participants synchronized the instructed action with the distractor movie, compared to when they synchronized the distractor action with the distractor movie. Although we still observed a significant bias in the latter condition, this finding indicates a degree of specificity in AI effects for the identity of the synchronized action. Overall, our data show that MI can substantially modulate the effects of AO on subsequent execution, wherein: (1) combined AO + MI can enhance AI effects relative to passive AO; (2) observed and imagined actions can be flexibly coordinated across different action types and planes; and (3) conflicting AO + MI can abolish AI effects. Therefore, combined AO + MI instructions should be considered in motor training and rehabilitation.

## INTRODUCTION

Research in action observation (AO) and imitation has made a series of important discoveries since the early 1990s. Traditional paradigms where imitation tasks were explicitly instructed are now complemented by research investigating a broader range of imitation-related phenomena (Heyes, 2013). For example, in naturalistic social settings, interacting partners often exhibit subtle but spontaneous mimicry of each other’s behavior, such as their postures, gestures, and speech, typically without knowing or intending to do so (Chartrand and van Baaren, 2009). Research investigating the neurocognitive mechanisms that underpin such imitative behavior essentially shows that watching another person’s action primes execution of similar actions in the observer (*visuomotor priming*; for a review see Vogt and Thomaschke, 2007). More recently, this phenomenon has been termed automatic imitation (AI; Heyes, 2011): a type of stimulus-response compatibility (SRC) effect, wherein observing a task-irrelevant action (distractor) facilitates the performance of similar actions and interferes with the performance of dissimilar actions. AI effects typically consist of differences between response initiation times for compatible vs. incompatible distractor actions (c.f., Brass et al., 2000, 2001; Stürmer et al., 2000). In addition, imitation biases can also be demonstrated using kinematic data. For instance, we recently quantified the magnitude of AI effects in the cycle time of compatible and incompatible rhythmical actions (that is, the “kinematic fidelity” of AI, Eaves et al., 2012). In the present research we investigated whether the magnitude of this imitation bias can be modulated by a range of motor imagery (MI) and execution instructions during distractor observation. Next we describe how our previous findings led us to instruct MI *during* AO in the present two experiments.

In our previous study (Eaves et al., 2012) participants saw a picture from a set of eight everyday rhythmical actions (“instructed action”). They then passively observed a short rhythmical distractor movie of either the same or a different action, before executing the instructed rhythmical action. Our subtle manipulation of distractor cycle times (slow or fast) produced a robust imitation bias in the cycle times of the participants’ subsequently executed actions, that is, towards the speed of the observed distractor. This bias was only a small fraction both of our modulations in the distractor speed, and of the modulations that participants could produce when intentionally imitating the distractor speeds. Relative to a fully compatible condition, the imitation bias was reduced equally (but still present) in three incompatible conditions, wherein instructed and distractor actions differed in (a) type (e.g., tooth brushing vs. window wiping), (b) plane of motion (horizontal vs. vertical), or (c) both. Accordingly, we found no evidence for separable (i.e., additive) contributions when both action type and plane were simultaneously incompatible. Instead, the distractor’s impact on motor processing was generally reduced whenever the observed action was not directly relevant to the observer’s intended actions. We conceptualized this finding further using Cisek and Kalaska’s (2010) framework of biased competition, and speculated that both the instructed and distractor actions can be modeled as parallel sensorimotor streams, which may or may not compete with one another.

In the present two studies, we employed the same extended (offline) SRC paradigm as in Eaves et al. (2012) and investigated the impact of different forms of MI during AO on this AI effect. The rationale for doing this was twofold. First, in our earlier study the compatibility between observed and executed actions had served to indirectly manipulate the competition between the two hypothetical sensorimotor streams. In the present studies, however, we used different MI instructions during AO (i.e., “AO + MI”) as more direct means of manipulation. Second, a series of recent studies have shown the neurophysiological impact of instructing MI during AO. When participants imagined performing the action that they simultaneously observed (i.e., AO + MI), a larger number of corticomotor regions were activated compared to AO (Macuga and Frey, 2012; Nedelko et al., 2012; Berends et al., 2013; Villiger et al., 2013). Stronger activations in motor and motor-related areas were also shown for AO + imitative execution, compared to both AO + MI and AO alone (Macuga and Frey, 2012; Villiger et al., 2013). While those authors suggested motor rehabilitation and training programs might be enhanced if practitioners combined AO + MI instructions, to our knowledge there is currently no behavioral evidence to demonstrate the effects of such instructions on overt motor behavior.

In Experiment 1 we contrasted two types of AO + MI instructions. During distractor AO, participants imagined from a first person perspective the physical sensation and effort involved in performing the instructed action in synchrony with the rhythmical distractor (AO + synchronized MI), or they imagined their own hand in the static start-posture needed for the instructed action (AO + static MI). By definition synchronized MI instructed tight temporal couplings between the parallel AO and MI simulations, while static MI required participants to effectively decouple their internal simulation of their own rigid hand posture from the on-going and dynamic AO sensorimotor stream.

In Experiment 1 our first aim (1.1) was to investigate if the imitation bias was stronger in later execution for synchronized MI compared to static MI. Our second aim (1.2) was to investigate if synchronized MI would significantly enhance the imitation bias relative to that which we obtained previously for passive AO (Eaves et al., 2012). Our third aim (1.3) was to assess if static MI would reduce the bias relative to this same passive AO condition. In Experiment 2 we pursued four additional aims. Our first aim (2.1) was to assess whether overtly executing an action and synchronizing this with the distractor action (AO + synchronized execution) would increase the imitation bias relative to AO + synchronized MI. In addition, we explored whether the imitation bias would be specific to later execution of the action that was synchronized with the distractor (i.e., the “distractor-synchronized action”), or if synchronization would also influence later execution of (aim 2.2) different action types, and (aim 2.3) in different planes of motion. Finally, our fourth aim (2.4) was to replicate the findings obtained for synchronized MI in Experiment 1. We report Experiment 1 first, and then describe the rationales for the aims of Experiment 2 in more detail. In summary, we pursued the following aims:

### Experiment 1

Is the imitation bias more pronounced for:

1.1. AO + synchronized MI compared to AO + static MI?
1.2. AO + synchronized MI compared to the imitation bias that we obtained previously for passive AO (Eaves et al., 2012)?
1.3. Passive AO compared to static AO?

### Experiment 2

Is the imitation bias:

2.1. increased for AO + synchronized execution, compared to AO + synchronized MI?
2.2. reduced when, during AO, participants imagined (or executed) an action different from the subsequently executed action, compared to imagining (or executing) the same action?
2.3. reduced when, during AO, participants imagined (or executed) the instructed action in a plane different from that of the subsequently executed action, compared to imagining (or executing) in the same plane?
2.4. In addition, we were seeking to replicate the findings for synchronized MI in Experiment 1, namely that there was no effect of action type compatibility (aim 2.4a), and that synchronized MI produced a stronger imitation bias than static MI (aim 2.4b).

## EXPERIMENT 1

### TASK AND DESIGN

On each trial participants observed a picture of a to-be-pantomimed rhythmical action (“instructed action”), followed by a short distractor movie. They then executed the instructed action. We studied actions that are typically performed relatively slow (“habitually slow actions”) as well as habitually fast actions. Within each habitual speed category, slow and fast versions of each distractor action were used.

Four blocks of thirty-two trials were conducted, with two blocks run on each consecutive day. A four-factorial repeated-measures design was used. MI content during distractor AO was manipulated across the two blocks run in each session (synchronized MI or static MI), in a counterbalanced order across participants. The other three factors were manipulated within each block of trials: habitual action speed (slow or fast), distractor speed (slow or fast), the compatibility between instructed and distractor actions, in terms of action type compatibility (same or different action: SA or DA), and dominant plane of motion compatibility (same or different plane: SP or DP). Combining the two individual compatibility manipulations yielded one compatibility factor with four levels: SA/SP, SA/DP, DA/SP, and DA/DP.

Note that the two factors of action type compatibility and plane compatibility were derived from pooling the data from their four constituent factors, namely: (1) instructed action type (face- or surface-oriented, see Materials and Methods), (2) instructed action plane (horizontal or vertical), (3) distractor action type (same or different), and (4) distractor action plane (same or different). The full combination of these four factors with habitual action speed and distractor speed resulted in 64 trials for both MI conditions, half of which were presented in a quasi-random order within each block on Day 1, and the other half on Day 2. As a result of the pooling, each cell of the effective four-factorial design consisted of an average across four trials.

We avoided two potential confounds that would have been associated with including a passive AO instruction in the present design. First, instructing both “passively observe” and “synchronize with” the distractor on consecutive and counterbalanced blocks in the same experiment could have encouraged active synchronization during passive AO trials. Second, an order effect would likely have been induced if all passive AO trials had been run at the start of each experiment. Therefore, we compared the data sets obtained from the present Experiments 1 and 2 to a passive AO data set that we collected previously (Eaves et al., 2012). While all three studies employed different instructions during AO, the cross-experiment comparison was equitable since all three experiments used the exact same trial structure and presented the same stimuli across trials for the same time periods. For a full description of all statistical analyses used, please see “Data Analysis.”

### RESULTS

**FIGURE 1:**
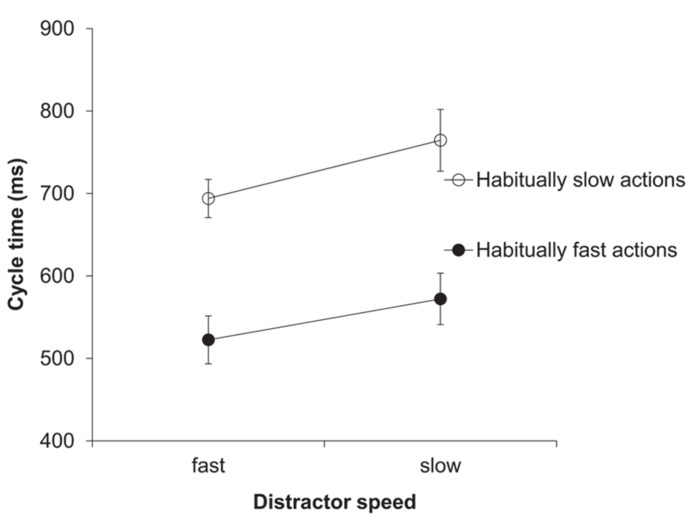
** Experiment 1: cycle times (ms)**. Mean cycle times for the factors habitual speed and distractor speed. Error bars show the standard error of the mean.

The two-factorial ANOVA on the cycle time (ms) data yielded a significant main effect of distractor speed, *F*(1,11) = 20.32, *p *= 0.001, $\eta_{p}^{2}$ = 0.65. As predicted, response cycle times were shorter after seeing a fast compared to a slow distractor (608 vs. 668 ms; see **Figure 1**). Trivially, the main effect of habitual speed was also significant, *F*(1,11) = 64.1, *p *< 0.001, $\eta_{p}^{2}$ = 0.85. The two-way interaction between distractor speed and habitual speed was not significant.

**FIGURE 2:**
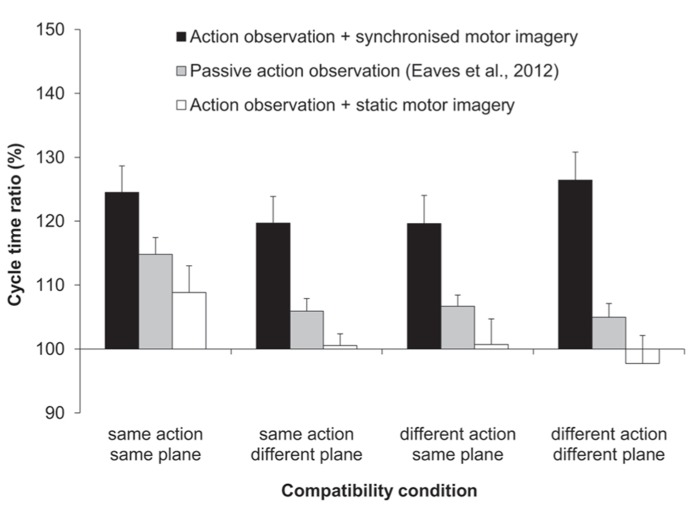
** Experiment 1: cycle time ratios (%)**. Mean cycle time ratios (with standard error of the mean) for three factors involved in Experiment 1: MI content, action type compatibility, and plane compatibility. Data obtained previously by Eaves et al. (2012) for passive-AO is also displayed. The cycle time ratio in the distractor actions was 150%.

The three-factorial ANOVA performed on the ratio data yielded only a significant main effect of MI content, *F*(1,11) = 16.67, *p *< 0.01, $\eta_{p}^{2}$ = 0.6 (see **Figure 2**). That is, the slow:fast ratio of response cycle times was significantly closer to that of the display (150%) following AO + synchronized MI (123%), compared to AO + static MI (102%). Both the main effects of compatibility and of habitual speed were not significant, and there were no significant interactions.

Running a series of simple effect analyses on the cycle time (ms) data for the synchronized MI condition confirmed that the main effect of distractor speed was significant in all four compatibility conditions (all *p*s ≤ 0.001, all $\eta_{p}^{2}$ s ≥ 0.62). In contrast, for the static MI condition, the main effect of distractor speed was not significant in each of the three incompatible conditions (all *p*s > 0.41, all $\eta_{p}^{2}$ s ≤ 0.06), and only approached significance for the fully-compatible SA/SP condition (*p* = 0.076, $\eta_{p}^{2}$ = 0.26).

Next we compared the imitation bias that we had obtained previously for passive AO (Eaves et al., 2012), to the bias obtained first for synchronized MI and second, for static MI. In both two-factorial, mixed measures ANOVAs the between-subjects factor was experiment (two levels) and the within-subjects factor was compatibility (three levels: SA/DP, DA/SP, and DA/DP). Note that the fully compatible SA/SP condition was excluded from both of these analyses. Since we previously submitted that participants could have covertly used the fully compatible “task-irrelevant” distractor as a strategic guide for their own actions, only the incompatible conditions in our previous experiment can be taken as evidence for genuine AI effects. The first of these two analyses compared passive AO to synchronized MI (see **Figure 2**). The main effect of experiment was significant, *F*(1,20) = 12.02, *p *< 0.01, $\eta_{p}^{2}$ = 0.38. Here the magnitude of the imitation bias was significantly stronger for the present synchronized MI condition (122%) compared to that for passive AO (106%). The main effect of compatibility and the two-way interaction between experiment and compatibility was not significant. The second of these two analyses compared passive AO to static MI. Here the main effect of experiment exhibited only a trend towards significance, *F*(1,20) = 2.86, *p *= 0.110, $\eta_{p}^{2}$ = 0.13, wherein the imitation bias was numerically reduced for static MI relative to passive AO (100 vs. 106%). The main effect of compatibility and the two-way interaction between experiment and compatibility was not significant.

### DISCUSSION

Regarding our first aim (1.1), synchronized MI significantly enhanced the magnitude of the imitation bias across all four compatibility conditions compared to static MI (123 vs. 102%, respectively). With regard to our second aim (1.2), the imitation bias was significantly more pronounced for the three incompatible synchronized MI conditions compared to the three incompatible passive AO conditions that we had previously studied (123 vs. 106%, Eaves et al., 2012). The most likely explanation is that synchronized MI increased the strength of sensorimotor coupling to the display. In essence, for synchronized MI we instructed participants to generate a dynamic internal motor simulation of the instructed action. This involved imagining the physical sensation and effort involved in performing this action. They then coupled the spatio-temporal features of this internal simulation with those of the second sensorimotor representation – that of the observed distractor action. Accordingly, this instruction enhanced (covert) sensorimotor coupling with the display, which biased later execution.

We further found that the imitation bias was equally strong across the four compatibility conditions for AO + synchronized MI. This indicates that similarly tight temporal couplings are possible between AO and MI, even when their contents do not match, with respect to the action type, plane of motion, or both. Accordingly, AO + synchronized MI appears to be a relatively flexible process that can accommodate a good range of AO + MI configurations.

These findings have direct importance for applied practitioners who wish to improve motor learning (e.g., in sports, pilates, yoga, and dance) and rehabilitation procedures (e.g., in stroke and neuro-degenerative patients). Practitioners typically regard both AO and MI as two potentially useful but distinctly separate adjunct tools (see Hodges et al., 2007; Braun et al., 2013; Poliakoff, 2013). However, our data provide the first behavioral evidence that combined AO + MI instructions can significantly modulate AI effects in the kinematics of later execution. Therefore, the present findings are in line with the recommendations of the recent neurophysiological studies (Macuga and Frey, 2012; Nedelko et al., 2012; Berends et al., 2013; Villiger et al., 2013), in that they support calls for new approaches to rehabilitation and training to include combined AO + MI instructions (see Vogt et al., 2013).

Regarding our third aim (1.3) we found that the imitation bias, which was clearly present following AO + synchronized MI, was largely absent after AO + static MI. For static MI there was no significant main effect of distractor speed in any of the four compatibility conditions (the numerically larger bias for the compatible distractors presumably indicates that it was slightly harder to remain decoupled from the display when the observed and imagined actions matched). In addition, we found a clear trend for a reduced imitation bias in static MI (100%) relative to passive AO (106%) in Eaves et al. (2012), where distractor speed effects were significant in each individual compatibility condition. Most likely, the absent imitation bias for the static MI condition is due to a dominant effect of MI (here: static) on subsequent motor execution, relative to the otherwise robust effect of AO. This result was not unexpected, given that participants were instructed to decouple their MI content from the concurrent AO process in the static MI condition.

Taken together the present data provide a first clear-cut demonstration of the strong modulatory effects of MI instructions during AO. While synchronized MI enhanced the imitation bias beyond passive AO, even under conditions where AO and MI contents were not identical, static MI practically abolished this AI effect. Our data also provide the first empirical support for a spectrum of different AO + MI states, ranging from congruent to coordinative, and finally to conflicting cases of AO + MI, as described next (for an extended account, see Vogt et al., 2013).

First, as in our Experiment 1, the neurophysiological experiments described in the Introduction used *congruent AO + MI* instructions: participants imagined themselves performing the same action that they simultaneously observed (c.f., Nedelko et al., 2012; Macuga and Frey, 2012). In our version of this task, we instructed participants to “switch on” their awareness of their own body schema and map the observed action onto their own felt hand (i.e., compatible synchronized MI). Subjectively this is different from simply observing, and our behavioral data clearly underpin this. A second AO + MI state is *coordinative AO* + *MI, *which was represented in the present design by the incompatible synchronized MI conditions. In contrast to congruent AO + MI, which directs a narrow focus of attention towards tight synchronization, coordinative AO + MI offers a potentially limitless array of spatio-temporal configurations between observed and imagined actions. Therefore, this arrangement invites many open questions and interesting lines of empirical enquiry (see Vogt et al., 2013). In rehabilitation, for example, patients might benefit from watching video-taped repetitions of a naturalistic action while making progressive changes in either range of motion or force production in their coordinated MI across trials. Most likely, both congruent and coordinative AO + MI states can be shaped into effective training procedures.

Finally, congruent and coordinative AO + MI states can be distinguished from cases of *conflicting AO* + *MI*, which we have implemented here via the static MI condition. While such conditions are unlikely beneficial for practitioners, they may prove an important research tool, similar to the use of compatible and incompatible visual stimuli in research on AI. It is also useful to contrast the present detrimental effect of static MI on AI with other proposed inhibitory mechanisms. In particular, previous research has identified that both AO and MI can give rise to motor commands, but that these are typically blocked at some level of the motor system by inhibitory mechanisms (e.g., Hardwick et al., 2012; and see Brass and Heyes, 2005; Guillot et al., 2012). At present, the processes by which this inhibition is achieved are not yet clear. For example, it is not clear whether inhibition is mediated by specific brain structures, or by intracortical facilitation/inhibition (Di Rienzo et al., 2013). In contrast, the present detrimental effects of static MI on AI most likely reflect a different class of “inhibitory” processing, namely a decoupling of the default impact of AO on motor processing by concurrent engagement in conflicting MI. For example, in an applied sporting context, MI of an action that differs from that of an observed opponent is one feasible strategy for avoiding an unwanted bias in later execution. Although this is presently a tentative suggestion, it may warrant further empirical investigation in the future.

Overall, Experiment 1 shows: (1) combined AO + MI instructions can enhance AI effects relative to passive AO; (2) AO and MI content can be flexibly coordinated across different planes and different action types; and (3) conflicting AO + MI content can abolish AI effects. Furthermore, we hope that the above classification of the three AO + MI states might motivate both applied and basic researchers to examine the boundaries, characteristics and opportunities for practical implementation of each state further. In Experiment 2 we explored two related themes. First, we studied the potentially stronger impact of motor execution, as compared to MI, during AO. Second, we manipulated the overlap between MI (and motor execution) content during AO with the subsequently executed action, in order to further explore the specificity of the imitation bias.

## EXPERIMENT 2

Since the largest gains in motor proficiency are most likely available through physical rather than mental practice (c.f., Higuchi et al., 2012), we wanted to assess the relative contributions of each. Therefore, in Experiment 2 our first aim (2.1) was to compare the magnitude of the imitation bias for AO + synchronized MI relative to that obtained from synchronizing overt motor execution with the display (AO + synchronized execution). For two reasons we expected an increase in the imitation bias for synchronized execution compared to synchronized MI. First, sensorimotor involvement should increase during synchronized execution (e.g., Macuga and Frey, 2012; Villiger et al., 2013). Second, temporary losses in synchronicity may be more frequent for synchronized MI, where afferent information is reduced. Given that there was no compatibility effect for synchronized MI in Experiment 1, we expected similar results for synchronized execution in Experiment 2.

In Experiment 2 we also studied the effects of a wider range of MI and overt synchronization instructions on subsequent execution. While in Experiment 1 the content of MI was always the to-be-executed (instructed) action, in Experiment 2 we used three different “synchronization type” instructions (i.e., for both MI and overt execution during AO, see aim 2.1). The first condition, “Synchronize the Instructed action” (SI), resembled the standard instructions in Experiment 1. Relative to this SI condition, we manipulated the extent of the overlap between the imagined (or overtly executed) action with the observed action in two ways. In the second condition, “Synchronize the Distractor action” (SD), participants imagined performing (or overtly performed) the action shown in the distractor movie. Independent of this manipulation, we again manipulated the compatibility between the instructed and distractor actions (manipulation of “action type compatibility” as in Experiment 1). This meant that, during observation of incompatible distractor actions, participants imagined (or executed) an action that was the same as the distractor, but different from the instructed action that they would later execute (e.g., while observing window wiping they imagined performing window wiping and then subsequently executed tooth brushing). Note that for compatible action types, the SI and SD conditions were identical.

The third synchronization instruction in Experiment 2 was “Synchronize the Instructed action in the Orthogonal Plane” (SIOP). Here participants imagined performing (or overtly performed) the instructed action but in the plane orthogonal to that shown in the distractor movie. Note that, unlike in Experiment 1, in Experiment 2 the dominant plane of motion was always compatible between the instructed and distractor actions, thus the plane of the synchronized action was always different from that of both the instructed and distractor actions. For a full overview of the experimental conditions in Experiment 2, see **Figure 4** and **Table 1**.

**Table 1:** Summary of the three synchronization instructions in Experiment 2.

Synchronization type	Instructed action (picture)	Distractor action (movie)	Action synchronized with distractor	Executed action
Synchronize Instructed action (SI)	Tooth brushing	Window wiping	Tooth brushing	Tooth brushing
Synchronize Distractor action (SD)	Tooth brushing	Window wiping	Window wiping	Tooth brushing
Synchronize Instructed action in the Orthogonal Plane (SIOP)	Tooth brushing	Window wiping	Orthogonal tooth brushing	Tooth brushing

*These instructions varied the degree of overlap between the action that was synchronized with the distractor and the subsequently executed instructed action. Within each synchronization type, participants were instructed to either imagine or overtly perform the required action during distractor observation. The table shows the instructions for one example trial involving incompatible actions.
*

The purpose of the SD and SIOP conditions was to assess if reducing the overlap between the distractor-synchronized action and the subsequently executed action would affect the imitation bias towards the observed rhythm, relative to the standard SI condition. Specifically, in the incompatible SD condition, we were interested if imagining (or performing) a given action with a certain distractor speed would also affect the speed of subsequently performing a different action (aim 2.2). A negative finding would demonstrate a highly action-specific priming effect, whereas a positive finding would demonstrate a degree of generalization for the observed rhythm across different imagined and executed actions.

Regarding our third aim (2.3) we assessed if imagining (or performing) a given action with a certain distractor speed would also affect the speed of subsequent execution in a different plane (i.e., compatible SIOP vs. compatible SI trials). A negative finding would indicate that the imitation bias was highly plane-specific, whereas a positive finding would demonstrate a degree of generalization of the imagined (or performed) rhythm across different planes of motion. Note that the manipulation of plane compatibility in our previous studies (Eaves et al., 2012, and Experiment 1 in the present paper) does not inform on this issue, since the previously imagined and to-be-performed planes of motion had always been identical. In addition to the two main aims regarding the manipulations of synchronization type (i.e., aims 2.2 and 2.3) we also studied if the possible plane-specific imitation effect might be further affected by a discrepancy between the observed and imagined (or performed) action. For example, it is conceivable that imagining vertical toothbrushing while observing horizontal tooth brushing affects the rhythm of subsequently performed horizontal toothbrushing (action-compatible SIOP condition), but also that the imitation bias might be weaker still when the MI needs to be synchronized with a movie of horizontal window wiping (action-incompatible SIOP condition, see **Table 1**).

Our fourth aim (2.4) for Experiment 2 was to replicate, via the SI condition, two findings from Experiment 1. Namely, can synchronized MI: (aim 2.4a) remove the action type compatibility effect as found in Eaves et al. (2012); and (aim 2.4b) enhance the imitation bias relative to both static MI and to our previous passive AO effects. Contrary to the SI condition, we predicted that action type compatibility would modulate the imitation bias in both the SD and the SIOP conditions. Therefore, we should also find a two-way interaction between synchronization type and action type compatibility. Within each synchronization type, we expected this finding to be pronounced similarly within each synchronization mode.

### TASK AND DESIGN

The same basic trial structure was used as in Experiment 1, whereby participants saw on each trial a picture of the instructed action, then a rhythmical distractor movie, and then executed the instructed rhythmical action. Unlike in Experiment 1, we kept the dominant plane of motion compatible between the instructed and distractor stimuli. Six blocks of thirty-two trials were conducted, with three blocks run on each of the two consecutive days. A five-factorial repeated-measures design was used. Across the three blocks run on each day participants followed one of three synchronization type instructions. First, as in Experiment 1, during distractor observation participants performed the instructed action type and synchronized this with the movie, before executing the instructed action (Synchronize Instructed action: SI). Second, in condition “Synchronize Distractor action” (SD) we instructed participants to perform the distractor action type and synchronize this with the movie before executing the instructed action type. Third, in condition “Synchronize Instructed action in the Orthogonal Plane” (SIOP) participants performed the instructed action type and synchronized this with the movie, but in the orthogonal plane to that of the distractor movie. For a summary of these conditions, please see **Table 1**.

Each of the three larger blocks described above were split into four mini-blocks of eight trials. Synchronization mode (synchronized MI or synchronized execution, see aim 2.1 above) was manipulated across consecutive mini-blocks in an alternating order, which was counterbalanced across participants. The other three factors were manipulated within each mini-block of trials: habitual action speed (slow or fast), action type compatibility between the instructed and distractor actions (same action or different action; SA or DA), and distractor speed (slow or fast).

As in Experiment 1, we pooled the data across the constituent factors, resulting in 64 trials for each of the three synchronization types, half of which were presented in a quasi-random order across the four mini-blocks in session one, and the other half in session two. Again, each cell of the effective five-factorial design consisted of an average across four trials.

### RESULTS

**FIGURE 3:**
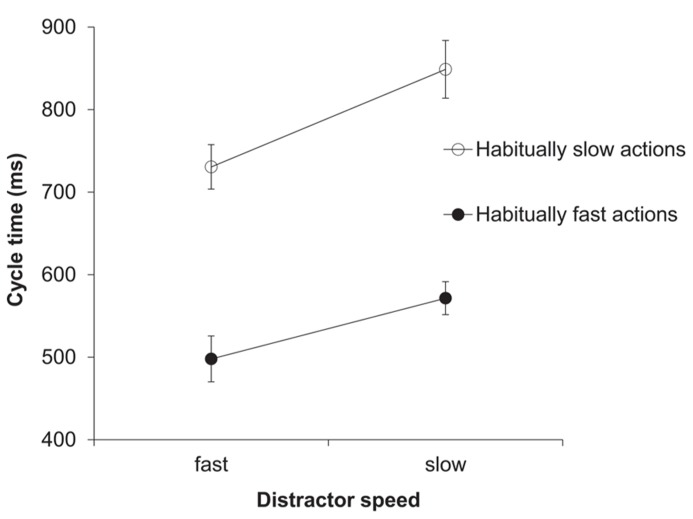
** Experiment 2: cycle times (ms)**. Mean cycle times for the factors habitual speed and distractor speed. Error bars show the standard error of the mean.

The two-factorial ANOVA on the cycle time (ms) data yielded a significant main effect of distractor speed, *F*(1,13) = 41.46, *p *< 0.001, $\eta_{p}^{2}$ = 0.76. As predicted, response cycle times were shorter after seeing a fast compared to a slow distractor (614 vs. 710 ms; see **Figure 3**). Trivially, the main effect of habitual speed was also significant, *F*(1,13) = 119.23, *p *< 0.001, $\eta_{p}^{2}$ = 0.9. The interaction between distractor speed and habitual speed was also significant, *F*(1,13) = 10.36, *p *< 0.01, $\eta_{p}^{2}$ = 0.44. This reflected the fact that, although the ratio of slow:fast distractor speeds was the same for each habitual speed (150%), the absolute difference between distractor cycle times was greater in habitually slow actions compared to habitually fast actions (see Data Analysis).

**FIGURE 4:**
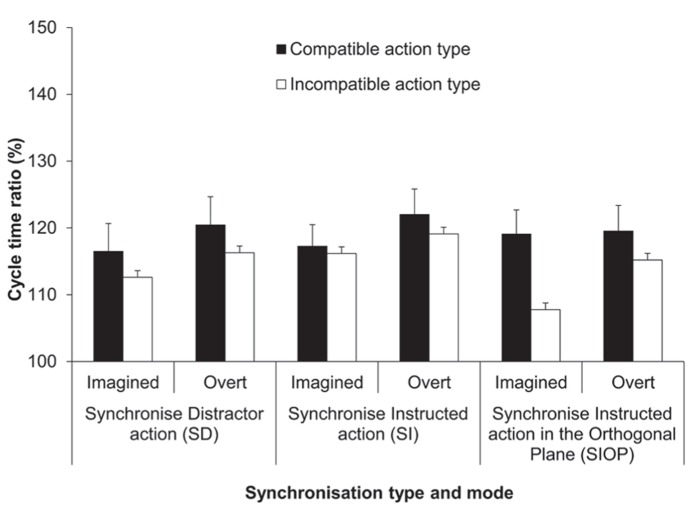
** Experiment 2: cycle time ratios (%)**. Mean cycle time ratios (with standard error of the mean) for the factors synchronization type, synchronization mode and action type compatibility. The SI condition (also used in Experiment 1) served as a reference condition for both the SD and SIOP conditions. The cycle time ratio in the distractor actions was 150%.

The four-factorial ANOVA on the cycle time ratio (%) data yielded a significant main effect of synchronization mode, *F*(1,13) = 12.38, *p *< 0.01, $\eta_{p}^{2}$ = 0.49 (see **Figure 4**). Overall, the imitation bias was more pronounced for synchronized execution compared to synchronized MI (119 vs. 115%, respectively). The main effect of synchronization type was also significant, *F*(2,23) = 3.4, *p = *0.05, $\eta_{p}^{2}$ = 0.21. Pairwise comparisons identified that the imitation bias was more pronounced for the SI condition compared to the SIOP condition (119 vs. 115%; *p* = 0.01), while these two conditions were not significantly different from the SD condition (117%; both *p*s > 0.05). The main effect of action type compatibility was significant, *F*(1,13) = 27.08, *p *< 0.001, $\eta_{p}^{2}$ = 0.68, wherein the response cycle time ratio was closer to the display ratio (150%) for compatible compared to incompatible action types (119 vs. 115 %). Different from the ANOVA on the mean cycle time data (ms), the effect of habitual speed was not significant in the cycle time ratios, confirming that the imitation bias was pronounced similarly for both habitual speeds, when expressed as cycle time ratios.

The only significant interaction was between synchronization type and action type compatibility, *F*(1.36,17.69) = 4.7, *p = *0.03, $\eta_{p}^{2}$ = 0.27. Pairwise comparisons showed that the imitation bias was significantly reduced when action type was incompatible compared to compatible between the instructed and synchronized action for both the SD (119 vs. 115%; *p *< 0.03) and the SIOP conditions (119 vs. 112%; *p *< 0.001), but not for the SI condition (120 vs. 118%; *p *> 0.05). A series of more specific simple main effect analyses compared compatible vs. incompatible action type within each synchronization mode for both the SD and SIOP conditions. These analyses confirmed that the bias was significantly reduced for incompatible compared to compatible trials within both modes for both the SD and SIOP conditions (all *p*s < 0.05). Additionally, when action type was compatible within both synchronization modes the bias was not modulated across the SI, SD, and SIOP conditions. First, these results confirm that in the SD condition the bias was reduced when the action type differed between the instructed and synchronized action for both synchronization modes. Second, in the SIOP condition the bias was not reduced when only plane was incompatible between these two actions. However, the bias was reduced when orthogonal synchronization was with an incompatible compared to a compatible action type within both synchronization modes (see **Figure 4**).

In two further analyses we compared the SI condition from Experiment 2 to the two MI conditions in Experiment 1. Both simple main effect analyses involved one between-subjects factor of experiment (two levels) and one within-subjects factor of action type compatibility (SA/SP or DA/SP). The latter reflected the fact that although four compatibility levels were used in both of our previous experiments, action type was the only compatibility factor manipulated in Experiment 2. The first analysis compared the present synchronized MI data (SI condition) to the equivalent synchronized MI data from Experiment 1. The main effects of experiment and of action type compatibility, as well as the related two-way interaction, were not significant. Therefore the magnitude of the bias for synchronized MI was replicated across Experiments 1 and 2. The second analysis compared the synchronized MI data from Experiment 2 (SI condition) to the static MI data from Experiment 1. The main effect of experiment was significant, *F*(1,24) = 9.5, *p < *0.01, $\eta_{p}^{2}$ = 0.28, wherein the imitation bias was greater for the present synchronized MI condition (117 vs. 105%, respectively). Again this replicated our earlier finding from Experiment 1. Unexpectedly, the main effect of action type compatibility was significant, *F*(1,24) = 5.11, *p = *0.03, $\eta_{p}^{2}$ = 0.18, wherein the bias was more pronounced for compatible compared to incompatible trials (113 vs. 108%), indicating a pocket of stronger distractor effects in a subset of the data. The two-way interaction between experiment and action type compatibility was not significant.

In two final steps we ran further simple main effect analyses. First, running these analyses on the cycle time (ms) data confirmed a significant main effect of distractor speed within each of the 12 conditions in Experiment 2 (all *p*s < 0.027, all $\eta_{p}^{2}$ s ≥ 0.32). Second, we then compared each of these twelve conditions to the fully-incompatible, and therefore most conservative, passive AO condition from our previous study (i.e., DA/DP, Eaves et al., 2012). The imitation bias for ten of the twelve conditions in Experiment 2 was significantly greater than that for passive AO (all *p*s ≤ 0.03). However, the bias was not significantly greater than passive AO for synchronized MI when action type was incompatible between the instructed and synchronized actions in both the SD and the SIOP condition.

### DISCUSSION

Regarding our first aim (2.1), the significant main effect of synchronization mode showed that the imitation bias was enhanced following overt compared to imagined distractor synchronization. This result was largely anticipated, since Macuga and Frey (2012) and Villiger et al. (2013) have already demonstrated stronger activations for AO + execution in a number of cortical sites. Here we provide the first behavioral evidence that nicely corresponds to this. It is most likely that the imitation bias was enhanced by increases in somatosensory feedback during overt compared to imagined execution. Accordingly, we can speculate that temporal losses in synchronicity are more common for AO + MI compared to AO + execution. This is interesting since a tight temporal coupling is typically found between an observed action and its internal motor representation (e.g., Borroni et al., 2005; see also Rizzolatti and Sinigaglia, 2010). Evidence from mental chronometry research has also established the principle of temporal congruency, whereby both MI and execution of the same action typically follow the same time course (see Collet et al., 2013). As such, more detailed studies are now required to examine the degree of spatio-temporal coupling between parallel internal simulations for different AO + MI configurations. Overall, the findings from the present two studies indicate that combined AO + MI instructions will be most useful in applied settings when overt execution is either restricted or not possible, for example, due to time or injury constraints. However, further research is needed to establish the efficacy of more specific instructions for practitioners.

Research using event-file paradigms has shown that when certain perception-action features co-occur during action observation (here: action type and rhythm), the neural signatures representing those particular features become closely associated (e.g., Nattkemper et al., 2010; Kühn et al., 2011). In our case, the execution or imagery of a given action should bias its subsequent execution towards the rhythm associated with it, whilst execution of an action without such association would not carry the same bias. Indeed we found that the imitation bias was present in all twelve compatibility conditions in Experiment 2. However, in relation to our second aim (2.2), both the main effect of action type compatibility and the two-way interaction between synchronization type and action type compatibility were significant. Simple main effect analyses showed that the imitation bias was significantly reduced for both execution modes in the SD condition when the instructed action type was incompatible compared to compatible with the distractor-synchronized action. Therefore, synchronization does not bias the execution of all subsequent actions to the same degree. Instead, our results highlight a degree of specificity for synchronization effects, whereby later execution is more strongly biased for action types that are represented during and, therefore, are directly involved in distractor synchronization. The absence of a compatibility effect within both modes for the SI condition is in line with this argument, since the instructed action was always represented during distractor synchronization in the SI condition. Here we replicate the result found for synchronized MI in Experiment 1 (see aim 2.4a), and show that the same trend exists for overt motor synchronization. Overall, although both imagined and overt actions can be flexibly coordinated with a range of observed actions that differ in planes and action types, this synchronization did not bias motor execution of a similarly broad range of action types. This was markedly so for the incompatible synchronized MI trials in the SD condition, where the imitation bias was no greater than that for our previously obtained passive AO condition (Eaves et al., 2012). 

Regarding our third aim (2.3), simple main effect analyses showed that synchronizing with the distractor in the opposite plane alone (i.e., compatible SIOP trials) did not affect the imitation bias relative to the standard SI condition. This result complements our earlier finding for the plane compatibility manipulation in Experiment 1 (for AO + synchronized MI). Together these results demonstrate that AO and MI contents can be flexibly coordinated across different planes of motion and, accordingly, that the AI effect as studied here does not rely on plane compatibility. Previous research has shown that when an actor performs a rhythmical arm movement while observing a spatially orthogonal rhythmical action, variability increases in the actor’s movements orthogonal to the instructed action (Kilner et al., 2003, 2007; Bouquet et al., 2007; Richardson et al., 2009; Romero et al., 2012). Accordingly, it is possible that a similar interference effect occurred during AO + synchronization in the present SIOP trials. However, the present design was not optimized to study such effects, and we instead used the cycle time of later execution to indicate the temporal coordination between the previously observed and synchronized actions. Here we demonstrate that, despite those possible increases in spatial variability, plane compatibility did not modulate the temporal coupling for both overt and imagined actions. This tentatively suggests that temporal couplings are relatively unaffected by spatial alignment.

While the compatible SIOP condition did not modulate the imitation bias relative to the compatible SI condition, we did find that the bias was reduced, although still present, for incompatible compared to compatible SIOP trials in both execution modes, that is, when participants synchronized in a different plane with a different distractor action. This result is in line with that for the incompatible vs. compatible SD trials. The action-incompatible SIOP trials thus identified a further boundary condition to the otherwise rather flexible coordination of AO and MI contents. This is not too surprising, given that this condition presented a considerable challenge to participants, in terms of the complexity of the task. This was primarily born out when synchronization was imagined rather than overt, which presumably reflected a reduced motor involvement for MI compared to execution. Overall, while the imitation bias was present in each of the 12 compatibility conditions of Experiment 2, the reduced bias found for the action-incompatible SD and SIOP conditions identifies a degree of specificity of AO + MI processes for the subsequently performed action.

With regard to our fourth aim (2.4b), Experiment 2 nicely replicated our finding from Experiment 1 that the imitation bias for synchronized MI was greater than that for both static MI (Experiment 1) and passive AO (Eaves et al., 2012). The imitation bias was also pronounced equally across Experiments 1 and 2. However, unlike in our previous analysis of synchronized MI vs. static MI in Experiment 1, which involved four compatibility levels, there was a main effect of compatibility when synchronized MI (Experiment 2) was compared to static MI (Experiment 1). Here the bias was stronger for compatible trials when only the SA/SP and DA/SP conditions were compared across the two experiments. This was likely due to the numerically greater distractor effects for compatible static MI (see **Figure 2**). However, in the main three-factorial ANOVA in Experiment 1 there was no main effect of compatibility or significant interaction involving compatibility and MI content, and since the simple main effect analyses in Experiment 1 revealed that static MI reduced the distractor effects in each compatibility condition (ms data), we refrain from further interpretation of this small pocket of significant findings in a subset of the data.

## GENERAL DISCUSSION

Foremost in the present data is that combined AO + MI instructions can facilitate AI effects in the cycle times of subsequently executed rhythmical actions. Therefore, our behavioral data support the calls for applied practitioners to include combined AO + MI instructions in motor training and rehabilitation programs. We also show that AO and MI content can be flexibly coordinated across different action types and different planes, and this can bias actions executed in either the same or orthogonal plane equally. We additionally show that static MI can practically abolish AI effects in later execution. 

While integrative accounts of AO and MI as sub-forms of action simulation are not new (see Shepard, 1984; Jeannerod, 1994, 2001, 2006), research efforts to study their contributions to action execution have largely branched out to focus on *either* AO *or* MI (see Vogt et al., 2013). Our paradigm represents a return to a more integrated approach to investigating these two closely related processes. As such, the data we obtained in Experiment 1 led us to distinguish three AO + MI states, ranging from congruent over coordinative to conflicting AO + MI states (Vogt et al., 2013). We hope these distinctions pave the way for both practitioners and experimental researchers to examine the boundaries, characteristics, and applied opportunities of each state further. Next we outline some considerations for future research in this area.

First, since congruent AO + MI instructions enhanced motor execution relative to passive AO, a major concern is that this strategy has seldom been accounted for in many of the existing AO experiments. In a large number of neuroimaging studies on AO, participants could have either covertly or spontaneously re-interpreted standard AO instructions as an AO + MI instruction. For example, it is not completely clear whether AO + MI was undertaken even on some “pure” AO trials in the four aforementioned imaging studies. Since we clearly show that combined AO + MI instructions can bias motor execution more strongly than passive AO, future studies should address this potential confound, wherein imaging data (in conjunction with behavioral measures) will be useful for more careful contrasts between pure AO, pure MI, and AO + MI content. Second, while the imitation bias was enhanced equally by both congruent and coordinative AO + MI in the present studies, future research could investigate more closely under which conditions superior training conditions might be afforded by one or the other AO + MI state. Third, we observed that during conflicting AO + MI (static MI in Experiment 1), the imitation bias was practically abolished. While this instruction will unlikely be beneficial for practitioners in the field, it may prove useful as a methodological tool, similar to the use of compatible and incompatible stimuli in research on AI. For example, this approach could address whether an inverse effect for AO on MI exists, wherein the resilience of MI to conflicting AO conditions is presently unknown.

We also showed that synchronising motor execution with AO produced a stronger imitation bias compared to AO + synchronized MI. This finding is in line with those neurophysiological studies showing greater motor cortical activations for AO + execution compared to both AO + MI and AO (Macuga and Frey, 2012; Villiger et al., 2013). Our behavioral data indicate that those increased motor activations could reflect increases in sensorimotor coupling processes. Overall, since AO + synchronized execution enhanced the bias further, AO + MI instructions appear best suited to applied settings when motor execution is either restricted or not possible, that is, due to time or injury constraints. 

Findings from event file paradigms (see Nattkemper et al., 2010) indicate that the co-occurrence of perception-action features would likely bias the execution of similar rhythmical actions. Indeed we found that the imitation bias was pronounced in all twelve compatibility conditions in Experiment 2. However, our findings for the incompatible SD and SIOP conditions highlight a degree of specificity for covert synchronization effects. Our data indicate that execution is biased more strongly by preceding sensorimotor processing when these two processes involve the same action. In comparison, disparity of plane between these two processes alone did not reduce the imitation bias. Therefore, although synchronizing motor processes (both MI and overt execution) with an observed action can accommodate a good range of configurations, we have also identified action disparity as a tentatively limiting factor.

Overall, Experiments 1 and 2 provide the first empirical evidence for the strong impact of different AO + MI states on AI effects in rhythmical actions. The distinction of the three AO + MI states now invites a range of new empirical and theoretical questions. For example, in which ways can we further assess the spatio-temporal couplings between parallel AO and MI streams, and what moderating roles might the sense of agency, MI perspective, and individual differences in motor expertise play? We believe the present work provides a valuable platform for addressing these issues further in an integrative way.

## MATERIALS AND METHODS

### EXPERIMENT 1

#### Participants

Twelve female participants (mean age 20.7 years; SD = 0.8 years) volunteered for the study. All had normal (*n* = 10) or corrected-to-normal vision. Participants were naïve to the study’s purpose, right-hand dominant, and without physical injuries. Written informed consent was obtained prior to participation, and ethical approval was granted by Lancaster University.

#### Stimuli and apparatus

**FIGURE 5:**
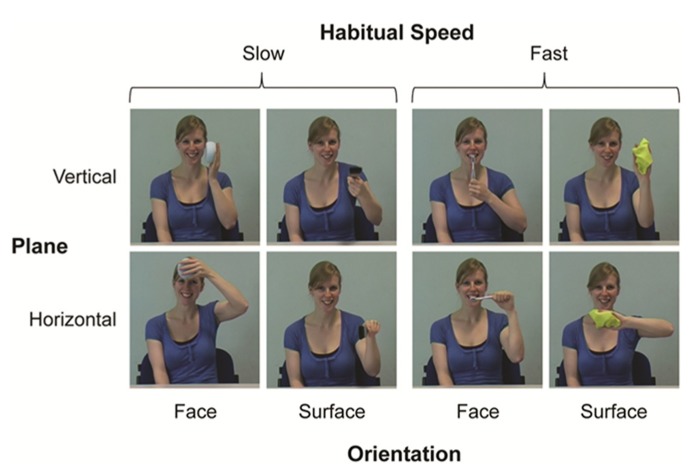
** Instructed action stimuli with the factors habitual speed, dominant plane of motion, and orientation (as used in** Eaves et al.,2012)

A conventional digital video camera (Panasonic NV-MX500B) was used to create the instructed action pictures and distractor movie stimuli. In total we used four different instructed actions. These were categorized either as actions that would typically be performed at a habitually slow pace (face washing: face-orientated; painting: surface-orientated) or fast pace (tooth brushing: face-orientated; window wiping: surface-orientated). Since each action was also instructed to be in either the horizontal or vertical plane, this gave a total of eight different instructed action pictures (**Figure 5**). The model performed all actions with her left hand to provide mirror images of the participants’ subsequent actions, who always executed actions with their right hand. This arrangement provided spatial compatibility between displayed and performed actions, which can facilitate imitation relative to an anatomically matched but spatially incompatible arrangement (e.g., Koski et al., 2003; Buccino et al., 2004).

Sixteen distractor movies were used in the main experiment, one slow and one fast version of each of the eight instructed actions. During filming, the model’s performance had been paced by a metronome to achieve the exact distractor speeds shown in **Table 2**, whereas throughout the experiment all stimuli were displayed without sound. Importantly, instructed action stimuli were always paired with a distractor stimulus from within the same habitual speed category. We used two habitual speeds for two reasons: first, we wanted to assess the imitation bias of the distractor movies on motor execution across a range of cycle times and not just for one speed. Second, the fact that participants executed, in quasi-random order, rhythmical actions with two substantially different habitual speeds served to divert their attention away from the more subtle manipulation of the distractor speeds. Finally, note that each instructed action was displayed with the relevant object (sponge, paintbrush, toothbrush, or cloth), which enabled quick discrimination between the actions, whereas participants performed pantomimed actions (i.e., without objects). The latter was done to avoid participants having to select the relevant object in the beginning of each trial. The distractor movies showed pantomimed actions to allow participants to better distinguish between instructed and distractor stimuli, and to potentially strengthen the impact of the distractor stimuli on their subsequent pantomimed execution.

**Table 2:** Distractor stimuli specifications.

Parameters	Habitually slow actions		Habitually fast actions	
Distractor speed	Slow	Fast	Slow	Fast
Beats per min	60	90	120	180
Cycle times (ms)	1000	667	500	333
Total cycles in 4 s	4	6	8	12
Slow:fast ratio (%)	150		150	

Participants sat at a wooden desk in a dimly lit room facing a 17-in LCD computer monitor (Apple Studio Display) positioned approx. 80 cm away from their head. All stimuli were displayed against a light gray background via PsyScript 2.3 software () running on a Power Macintosh G4 computer fitted with a digital I/O board. The start location for the participants’ right index finger and thumb was on an electro-conductive plate mounted on top of a 23 cm-tall wooden post, 20 cm in front of them on the desk. A magnetic motion sensor was fitted to the distal end of the second metacarpal bone of the right hand. Participants’ kinematic data were sampled at 103 Hz in 3-D space for 4 s periods using a Minibird Magnetic Tracking System (Ascension Technology Corp.), and stored on a separate PC. At the end of each trial, kinematic data plots were displayed on a second monitor, unseen by participants.

#### Procedures

***Familiarization.In*** Phase 1 participants learned to pantomime each action from a set of eight familiarization movies (eight actions with two attempts each). These movies were identical to the movies in the main experiment, except that the cycle times were mid-way between the distractor speeds shown in **Table 2**, that is, 75 bpm for the habitually slow actions, and 150 bpm for the habitually fast actions. Participants were given verbal feedback about their movement based on the kinematic plots visible to the experimenter. This ensured that their movement amplitude and cycle time aligned closely with the medium-paced stimuli. In Phase 2, participants saw a picture of each action while simultaneously pantomiming the same action for 4 s (16 trials). In Phase 3, they experienced the structure of trials for the main experiment, including the four compatibility conditions (16 trials).

In Phase 4, participants repeated Phase 3 but performed MI during AO. During distractor AO, participants imagined from a first person perspective the physical sensation and effort involved in either (1) performing a dynamic version of the instructed action that was time-synchronized with the rhythmical distractor (AO + synchronized MI), or (2) adopting the static start-posture needed for the instructed action (AO + static MI). Compatible and incompatible MI content was practiced in both conditions, and overt movements were only executed after distractor offset. In Phases 2 to 4, verbal feedback was only given if movements occasionally drifted away from the criterion amplitude (10 cm for all actions) or cycle times. Short versions of this familiarization procedure were run on each new day of testing.

**FIGURE 6:**
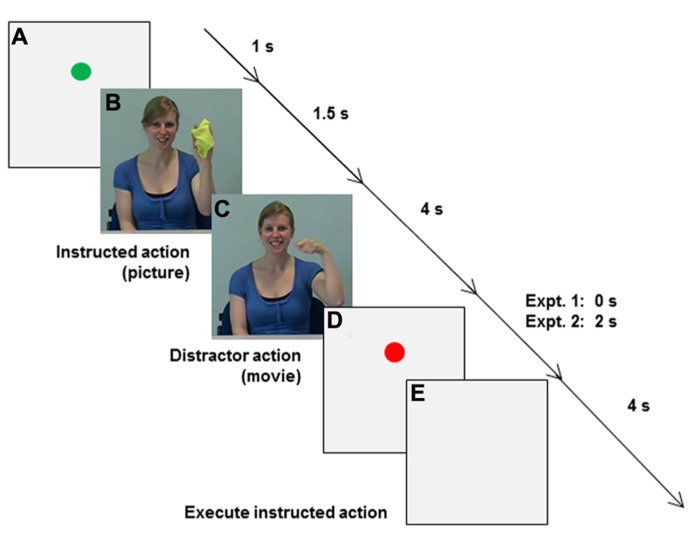
** Sequence of events in Experiments 1 and 2. (A)** A green circle appeared when participants placed their fingers in the start location. **(B)** A picture of the to-be-pantomimed instructed action was shown, followed by **(C)** a distractor movie of the model pantomiming either the same or a different action. During AO **(C)** in Experiment 1 participants engaged in either synchronized MI or static MI. In Experiment 2 synchronization type was manipulated during AO [Synchronize the Instructed action (SI), Synchronize the Distractor action (SD), or Synchronize the Instructed action in the Orthogonal Plane (SIOP) with the distractor action], as was synchronization mode (synchronized MI or synchronized execution). For Experiment 2 only, a pause was inserted **(D**: red dot) to separate synchronized actions **(C)** from **(E)** later execution of the instructed action, which was cued by the appearance of a neutral, light-gray background.

***Main experiment.*** When participants placed their fingers in the start location, a green circle appeared on the monitor for 1 s to mark the beginning of a trial (Event A in **Figure 6**). (B) Then a picture of the to-be-pantomimed “instructed action” was shown for 1.5 s, followed by (C) a distractor movie of the same girl pantomiming either the same or a different action for 4 s. During distractor observation participants engaged in either synchronized MI or static MI, while visually fixating on the model’s left eye, rather than directly coupling their vision to the model’s rhythmical arm movements (c.f., Schmidt et al., 2007; Eaves et al., 2012). (D) For Experiment 2 only, a pause was inserted (red dot) to separate synchronized execution from (E) execution of the instructed action, which was cued by the appearance of a neutral, light-gray background. The end of the 4 s kinematic recording interval (E) was indicated by a computer-generated auditory signal, after which participants were sometimes asked to verbally report distractor characteristics (see below) before moving their hand back to the start location.

In both Experiments 1 and 2 the core manipulation across trials was that of distractor speed, with a ratio of slow:fast movements of 150% (see **Table 2**). Participants were not informed of the distractor speed changes, and this manipulation was further concealed by the more prominent differences between the two habitual speeds across trials. To focus their attention on the distractor movie, participants in both experiments were asked to verbally recall the distractor properties (action type and dominant plane of motion) at the end of approximately 10% of trials. In both experiments testing was distributed over two consecutive days to reduce the possibility of physical fatigue. All blocks of trials were preceded by a single warm-up trial and interspersed by short rest periods.

### EXPERIMENT 2

#### Participants

Fourteen new participants (6 male, mean age 24.1 years; SD = 7.6 years) volunteered for the study. All had normal (*n* = 11) or corrected-to-normal vision. Participants were naïve to the study’s purpose, right-hand dominant (Edinburgh Handedness Inventory: *M* = 74; Oldfield, 1971), and without physical injuries. Written informed consent was obtained prior to participation, and ethical approval had been granted by Teesside University.

#### Stimuli and apparatus

The experimental setup and stimuli replicated those used in Experiment 1.

#### Procedures

***Familiarization.*** Phases 1–3 were identical to those used in Experiment 1, and Phase 4 was extended. Since learning is a key component of the PETTLEP model for mental imagery training (Holmes and Collins, 2001), participants completed the Movement Imagery Questionnaire-3 (MIQ-3; Williams et al., 2012) prior to participating. They executed overt followed by imagined actions and then self-reported the vividness of their experiences on three subscales: visual internal, visual external and kinesthetic imagery (12 actions in total; mean scores = 5.4, 5.3, and 5.1/7, respectively). On each day of testing, an MI script based on the PETTLEP principles was read out, instructing participants to engage in internal, first person kinesthetic MI of the instructed actions (one habitually fast and one habitually slow). They then practiced the two synchronization modes (synchronized MI and synchronized execution: 4–8 trials) for each synchronization type (SI, SD, and SIOP see below) ahead of the related block of trials in the main experiment.

***Main experiment.*** The trial structure is described in **Figure 6**. Participants were instructed to synchronize different actions with the distractor action. Instructions varied across blocks of trials as follows: (1) as in Experiment 1, participants Synchronized the Instructed action (SI) with the distractor movie; or (2) Synchronized the Distractor action (SD) with the distractor movie; or (3) Synchronized the Instructed action with the movie, but in the orthogonal plane (SIOP). Within each of the three conditions participants alternated between execution modes (synchronized MI or synchronized execution) across mini-blocks of eight trials. For synchronized execution, participants resumed the start position at distractor offset, wherein a red dot appeared for 2 s. This separated the distractor-synchronized action from the subsequently executed instructed action, wherein 3-D kinematics were tracked for 4 s.

***Data analysis.*** In both Experiments mean cycle times (ms) were calculated between peak minimum kinematic positions using a customized signal processing application created in Microsoft Visual Studio. First, a 6 Hz low-pass, second order, bi-directional Butterworth Filter smoothed the data. For both horizontal and vertical actions, the first data point taken was the first peak minimum of the first movement cycle. This avoided analyzing hand movements during the initial spatial positioning phase for each action before a stable workspace was reached. Mean cycle time was calculated across all peak minimum positions available within a 2 s time window. Typically this involved either two or three cycles for habitually slow actions and four or five cycles for habitually fast actions. All trials with erroneous responses (incorrect or no action) were discarded (Experiment 1: *n* = 10; Experiment 2: *n* = 63).

The two main dependent measures used in Experiments 1 and 2 were the mean cycle time (ms) and the ratio (%) between slow and fast distractor trials. While the absolute difference between distractor cycle times was greater in the habitually slow actions (667 vs. 1000 ms) compared to the habitually fast actions (333 vs. 500 ms), the ratio of slow:fast distractor speeds was the same for each habitual speed (150%). For economy of exposition in both experiments, we restricted the analysis of the mean cycle time data to two factors of interest. We then analyzed the additional factors involved in each experiment using the cycle time ratios. Accordingly, the mean cycle times (ms) were analysed in both experiments using a two-factorial, repeated-measures ANOVA with the within-subjects factors of distractor speed (only available for this measure) and habitual speed. In Experiments 1 and 2 the cycle time ratios (%) were subjected to three- and four-factorial repeated-measures ANOVAs, respectively. The within-subjects factors involved in Experiment 1 were MI content, habitual speed, and compatibility (four levels). In Experiment 2 the four factors were synchronization mode, synchronization type, habitual speed, and action type compatibility (two levels).

We then used a pair of two-factorial mixed measures ANOVAs to compare the imitation bias that we obtained previously for passive AO (Eaves et al., 2012), to that which we obtained first for synchronized MI and second for static MI in Experiment 1. We then used similar analyses to compare these latter two conditions to the synchronized MI data from Experiment 2. In a final step, we used a series of simple main effect analyses to individually compare all 12 conditions in Experiment 2 to the fully-incompatible, and therefore most conservative, passive AO condition from our previous study (Eaves et al., 2012).

All analyses were conducted using SPSS Statistics 21 (IBM). Where appropriate, these were adjusted for any violation of the homogeneity of variance assumption using the Greenhouse–Geisser correction. Alpha levels were set to 0.05, and effect sizes were calculated as partial eta squared values ($\eta_{p}^{2}$ ). To reduce type I error rates, Fisher’s least significant difference (LSD) contrasts were used in all pairwise comparisons, since four or less conditions were involved in each comparison (see Carmer and Swanson, 1973). 

Reaction time data were also recorded to identify trials with anticipatory (<200 ms; Expt. 1: *n* = 7; Expt. 2: *n* = 16) or omission errors (>1300 ms; Expt. 1: *n* = 2; Expt. 2: *n* = 7), which were discarded from all analyses. In total, 1.2% (Expt. 1) and 4.2% (Expt. 2) of all trials recorded were removed from the analyses.

## Conflict of Interest Statement

The authors declare that the research was conducted in the absence of any commercial or financial relationships that could be construed as a potential conflict of interest.
